# The Needle Anxiety Program: A Patient-Centered Initiative for Individuals With Developmental Disabilities

**DOI:** 10.7759/cureus.42253

**Published:** 2023-07-21

**Authors:** Julianna Rava, Kashia A Rosenau, Kendal Wilkie, Jessica Bernacki, Eric Curcio, Alice Kuo

**Affiliations:** 1 Medicine, University of California Los Angeles David Geffen School of Medicine, Los Angeles, USA; 2 Internal Medicine, University of California Los Angeles David Geffen School of Medicine, Los Angeles, USA; 3 Medicine-Pediatrics, University of California Los Angeles David Geffen School of Medicine, Los Angeles, USA

**Keywords:** needle phobia, developmental disabilities, patient-centered, needle anxiety, autism

## Abstract

Objective

To describe the development process of a patient-centered initiative focused on improving primary care health outcomes of patients with intellectual and developmental disabilities (IDD) and needle-related anxiety using evidence-based practices and novel approaches that can be implemented in outpatient settings. The overall outcome of the program is to increase vaccine uptake and accessibility in the IDD population as well as improve needle-related procedures in primary care settings to be more humane and effective.

Methods

The development process occurred in the context of a large healthcare system serving a diverse patient population in the U.S. and was led by an expert committee made of an multidisciplinary team of physicians, psychologists, ambulatory and clinic nurses, pharmacists, and anesthesiologists committed to promoting quality healthcare for the IDD population. Committee members were recruited within the healthcare system based on their relevant expertise. The methodology included an iterative and collaborative process that took place over three development phases: ideation and design, literature review and synthesis, and expert engagement. The ideation and design phase included a series of planning meetings among the expert committee, in which committee members identified preliminary concerns based on their expertise in the field and background knowledge on the current procedures related to improving routine care for individuals with IDD and/or needle-related anxiety. The literature review and synthesis phase led to the development of an annotated bibliography of research and clinical guidelines that synthesized findings on needle anxiety in clinical care. The expert engagement phase included all Committee members meeting for a final discussion to establish a tiered approach to utilizing evidence-based strategies that could be implemented across clinics within the healthcare system.

Results

The multidisciplinary team of experts developed a three-tier system, deployed sequentially as needed. The first tier focuses on training nurses in evidence-based behavioral modification strategies to implement as standard of care. The second tier uses the addition of a distraction device and topical analgesic to reduce anxiety in patients with slightly elevated procedural anxiety. The third tier involves a novel minimal sedation protocol using intranasal midazolam for patients with needle phobia that can be administered in an outpatient setting.

Conclusion

The Needle Anxiety Program eases the administration of needle-related medical procedures in the primary care setting for patients with IDD and needle-related anxiety. The use of evidence-based practices and a novel minimal sedation protocol for individuals with needle phobia assists in the completion of routine healthcare procedures, such as vaccinations and phlebotomy, in a patient-preferred setting. The purpose of delineating needle-related processes and procedures through the Needle Anxiety Program is to reduce health disparities for patients with IDD and promote uptake of the Needle Anxiety Program in similar healthcare settings.

## Introduction

Children and adults with intellectual and developmental disabilities (IDD) experience significant health disparities compared to the general population. Specifically, people with IDD are at increased risk for mental and physical health challenges - such as depression, anxiety, and cardiovascular, gastrointestinal, and metabolic issues [1-3]. During the COVID-19 pandemic, a diagnosis of IDD was associated with higher diagnosis and mortality rates of COVID-19 [4]. The compounding effects of these health issues have a significant impact on people with IDD, leading to a lower life expectancy for this population [3,5,6].

Further driving health disparities for people with IDD is low healthcare utilization rates [7]. Many of the co-occurring health challenges for people with IDD require routine needle-related medical procedures (e.g. vaccinations, blood draws) at a primary healthcare setting. However, many individuals with IDD experience needle-related anxiety that hinders the utilization of procedural medical care. People with IDD often experience greater levels of pain and sensory stimulation that make needle-related procedures intolerable [8,9]. Forcing the uptake of needle-related procedures can be traumatizing for an individual, leading to avoidance of routine healthcare in the future. On the other hand, when healthcare providers do not want to inflict undue stress on a patient with IDD and needle-related anxiety, they may skip the procedure entirely. This puts the patient at increased risk for more severe health complications in the long term. Consequently, addressing needle-related anxiety is crucial to improving the overall quality of life for people with IDD.

Needle-related procedural anxiety

Needle-related procedural anxiety is a common occurrence and one that negatively affects individuals’ completion of important preventive medical care [10-12]. The severity of needle-related anxiety varies - many people have a general fear of needles that requires additional strategies to alleviate discomfort during procedural care, while others have an extreme fear of needles that prevents the use of needles during medical care. Needle phobia, also known as trypanophobia or blood-injection-injury phobia, is the intense fear of injections, transfusions, blood, or other medical care related to needles that results in clinically significant avoidant behavior of needles [13]. While prevalence rates vary, it is estimated that approximately 10% of the general population experiences a fear of needles and 1-2% have needle phobia [14]. Among children, these rates are greater, with 15-20% of children experiencing anxiety towards needles and nearly 5% having a specific needle phobia [14,15]. Children and adults with IDD are nearly twice as likely to have needle-related anxiety compared to their peers [16-19]. Individuals with IDD already experience disparities in quality healthcare, therefore, addressing needle-related anxiety among this population is one opportunity for primary care providers to close the gap in health outcomes.

Current strategies to improve routine care for individuals with needle-related anxiety

Evidence-based practices to improve the uptake of medical care for individuals with IDD include behavioral interventions such as contingent reinforcement of compliant behavior, and/or absence of escape complemented with graduated exposure [12, 20-24]. Additional effective strategies include modeling, systematic desensitization, and cognitive behavior therapy [20,25-27]. To address pain management, providers deliver a topical anesthetic, such as a Eutectic Mixture of Local Anesthetics (EMLA) cream [28]. Distraction techniques are also used, such as a commercially-available nonpharmacological device that combines cold and vibration against the skin (Buzzy®, Pain Care Labs, Atlanta, USA), and having patients watch a video during the procedure [29-31]. While many of these intervention strategies are helpful, an integrated, comprehensive, systematic approach to applying these practices is needed to scale and offer them to the IDD community. Further, there is a subpopulation of individuals with IDD and needle phobia who have difficulty completing needle-related procedures even when evidence-based approaches are used. Therefore, novel approaches within primary care settings are needed to support the completion of routine procedures for all individuals with IDD and needle phobia. 

Primary care providers may consider strategies that have been successful in other areas of medicine to treat individuals with IDD and needle phobia. The American Society of Anesthesiologists Committee and the American Dental Association (ADA) established guidelines on mild to moderate sedation in outpatient settings [32,33]. Specifically, minimal sedation techniques (also known as procedural sedation) have been shown as an effective strategy to perform dental procedures for pediatric patients [34-36]. So far, these procedures have only been adapted to help individuals with IDD and/or individuals with severe needle-related anxiety complete routine care procedures in hospital settings [28]. However, routine care delivered in a hospital setting requires substantially more cost, time, and resources than care that can be delivered in the patient-preferred, outpatient setting.

Initiative aims

The aim of this paper is to present the design and lessons learned in developing a patient-centered needle anxiety protocol in a primary care setting. The overall goal of this process is to close the health equity gap for children and adults with IDD and/or needle-related anxiety by removing barriers to routine needle-related medical procedures in outpatient facilities.

## Materials and methods

Expert committee

The development of the Needle Anxiety Program involved a collaborative process among a multidisciplinary team of internal medicine physicians, a psychologist, clinic nursing staff, pharmacists, the ambulatory nursing team, and anesthesiologists. Committee members were identified and recruited to participate as experts with academic positions and research and/or clinical experience caring for people with IDD and/or needle-related procedural anxiety within the healthcare system. As Committee members were recruited for this process, they were informed about the general aims of this initiative. The Committee consisted of approximately 12 experts throughout the entire development process.

Methodology

The program’s development process used a modified Delphi method that consisted of three phases [37]: (1) Ideation and Design, (2) Literature Review and Synthesis, and (3) Expert Discussion.

Phase 1 (Ideation and Design)

The initial phase included a series of planning meetings among the expert committee, in which committee members identified preliminary concerns based on their expertise in the field and background knowledge of the current procedures related to improving routine care for individuals with IDD and/or needle-related anxiety. Through these planning meetings, the project objective was defined: to increase the uptake of needle-related procedures in an outpatient setting for patients with IDD that experience needle-related anxiety. Additionally, Committee members established a consensus method for meeting the project’s goals. It was decided that members of the academic clinical team would perform a literature review and provide a summary of the current strategies used to alleviate needle-related anxiety in primary care. Committee members would have an opportunity to review the findings, vote, and provide feedback asynchronously through a web-based survey. Committee feedback would be synthesized to help facilitate an expert discussion on how to create a program that meets the initiative's objectives. After the expert discussion, a second vote would take place and would be used in establishing the structure of the program.

Phase 2 (Literature Review and Synthesis)

The second phase led to the development of an annotated bibliography of research and clinical guidelines that synthesized findings into a literature review on needle anxiety in clinical care. The literature search involved a search of the PubMed and Web of Science databases for pilot and clinical studies, clinical guidelines, and systematic reviews published within the last 10 years (2010-2020). The following combined search terms were used: autism, developmental disabilities, intellectual disability, needle anxiety, procedural anxiety, needle phobia, trypanophobia**,** primary care, outpatient, intervention. Studies reporting interventions and pain management related to needle procedures were included. There were a limited number of papers focused on individuals with IDD and needle-related anxiety, therefore the literature review included studies, reviews, and guidelines focused broadly on needle anxiety interventions. Committee members were given time to review the material and provide feedback using a web-based survey that included open-text comment boxes for voting. Members voted on established intervention strategies and guidelines as well as anecdotal clinical techniques used for reducing needle-related anxiety during clinic visits. The survey responses were condensed to a list of strategies supported by the majority of Committee members.

Phase 3 (Expert Discussion)

The final phase included all Committee members meeting for a final discussion to identify priority evidence-based strategies that could be implemented across clinics. Members also discussed innovative strategies that have been effective in settings outside of primary care and anecdotal evidence presented by physicians and nurses on behalf of their patients with IDD and their families. The Committee members deliberated the structure of the program and the priority practices to be utilized at different stages of the program. The techniques most frequently endorsed by Committee experts were identified for the Needle Anxiety Program.

## Results

The program development process took 10 months from conceptualization to implementation (September 2019-July 2020); it resulted in a three-tier program incorporating both evidence-based practices and novel techniques. The overall goal of the program is to increase completion of vaccinations and other needle-related procedures by eliminating barriers due to needle-related anxiety among individuals with IDD in the primary care setting.

Needle Anxiety Program: three-tier system

**Figure 1:**
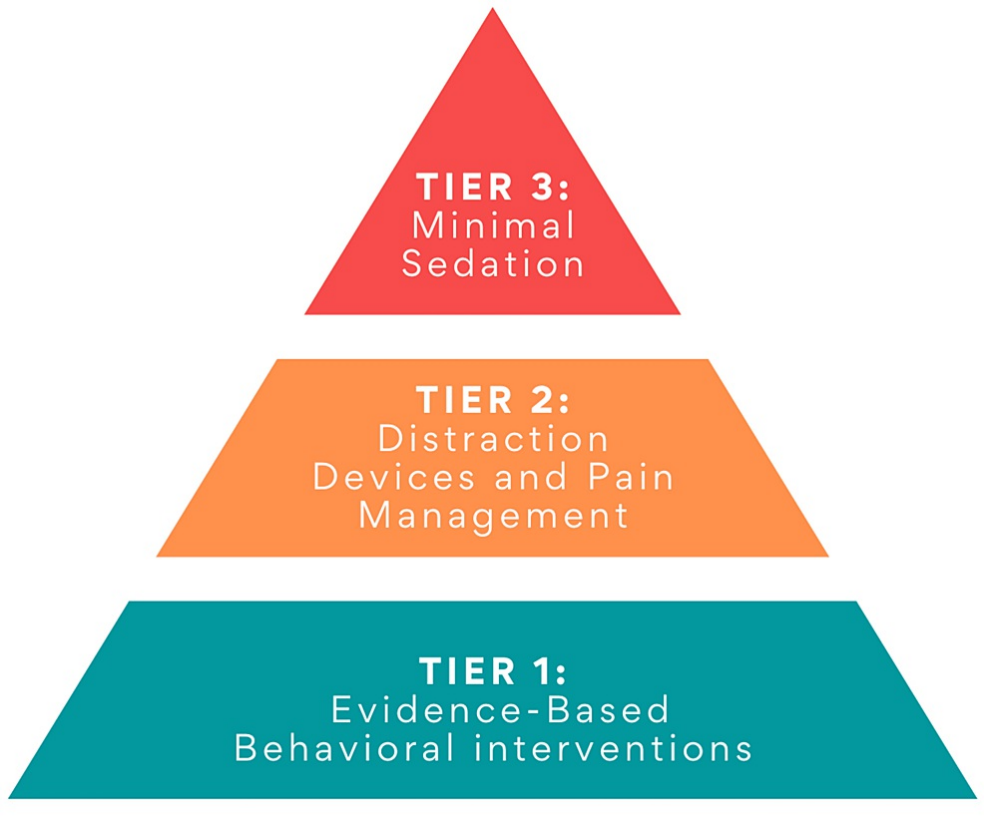
Needle Anxiety Program Three Tier System of Care

The needle-related anxiety spectrum can be addressed through a three-tier system (Figure 1): Tier 1) Evidence-Based Behavioral Interventions, Tier 2) Distraction Devices & Pain Management, Tier 3) Minimal Sedation. Among those experiencing needle-related anxiety, roughly 90% will use Tier 1 approaches, approximately 8% will need Tier 2 interventions, and 1-2% will need Tier 3 to complete needle-related procedures. For patients with IDD, we expect slightly more individuals to utilize Tier 2 and Tier 3 strategies. Thus, most patients with needle-related anxiety will require only Tier 1 or Tier 2 techniques, which are noninvasive, low-risk, evidence-based practices to reduce distress and discomfort during a medical care visit. Tiers 1 & 2 are designed to be administered by medical assistants, licensed vocational nurses, and registered nurses (RN). A small portion of individuals will need Tier 3, a novel minimal sedation protocol to be used in outpatient settings [38]. Tier 3 can be administered by a physician, RN, or nurse practitioner. 

Tier 1: Evidence-based behavioral interventions

The first tier focuses on training nurses and other clinic staff to deliver evidence-based behavioral strategies as part of routine clinical care for patients, emphasizing the needs of individuals with IDD and/or needle-related anxiety. Nurses were provided in-person or online training via a one-hour seminar presented by a pediatric psychologist. This training taught strategies for reducing distress and discomfort for patients during vaccinations and needle-related procedures. Evidence-based behavioral techniques included comfort positioning, distraction, and caregiver coaching [23,26].

Tier 2: Distraction devices and pain management

Tier 2 is initiated when Tier 1 techniques are not sufficient to reduce distress and complete the needle procedure. Strategies include pre-visit resource sharing, distraction devices (videos, Buzzy®), and an EMLA (numbing) cream. Prior to the procedure, the patient or family is provided with a list of evidence-based resources that educate patients on how to prepare for the upcoming needle procedure (e.g. Stanford Medicine Children’s Health pain management video guide [39]). At the appointment, patients are offered several distraction techniques: watching a preferred video during the medical procedure, mechanical stimulation (e.g., Buzzy®), and/or a topical analgesic, such as EMLA, to reduce the painful sensation caused by the needle procedure. The distraction techniques may be used congruently or alone. A Tier 2 approach often requires additional time built into the appointment for the topical analgesic activation and the preparation and use of other distraction devices. Tier 2 strategies should be used by trained healthcare professionals in coordination with the Tier 1 interventions.

Tier 3: Minimal sedation

**Figure 2:**
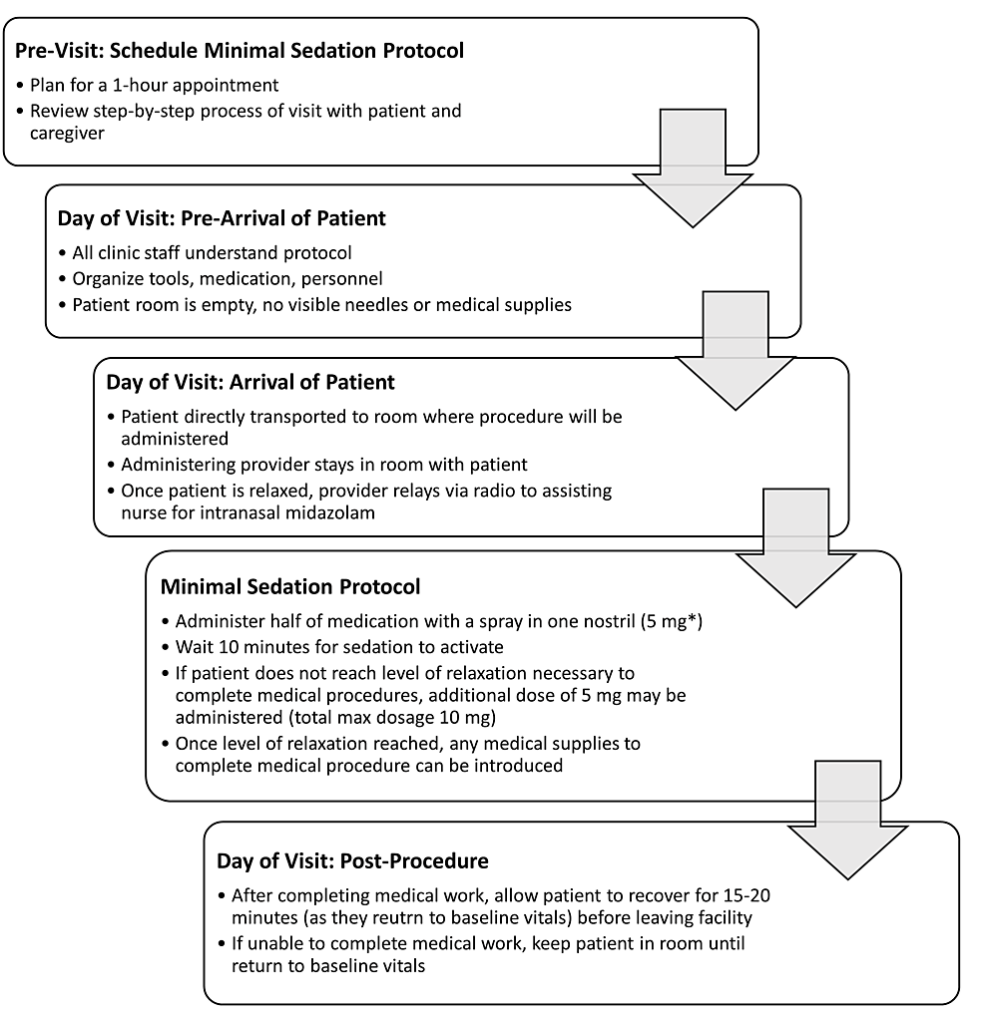
Appointment Instructions for Healthcare Providers Performing the Minimal Sedation Protocol *The recommended initial dose for older children and adults is a fixed dose of 5 mg of midazolam. For younger and smaller children, a dosage of 0.2 mg/kg is recommended. A total max dosage is 10 mg of midazolam [31].

Tier 3, minimal sedation, is recommended for individuals that had unsuccessful attempts completing medical procedures using the intervention strategies in Tier 1 and Tier 2 or were identified as appropriate candidates for Tier 3 by a physician. The minimal sedation protocol allows for a maximum of 10 mg of intranasal benzodiazepine (midazolam) for the intervention. Prior to the scheduled minimal sedation visit, the provider should explain the step-by-step process of the visit to the patient and their caregiver, including counseling on how the midazolam will be administered intranasally. Figure 2 includes detailed instructions of a scheduled minimal sedation visit to complete needle-related medical procedures.

Patients should not take any premedication prior to coming into the clinic for their appointment, as it can lead to oversedation. For some patients that take additional anxiolytic medication, particularly other benzodiazepines such as lorazepam, the advising physician must assess the patient prior to initiating Tier 3 protocol. For our clinic, approval of RN administration of the minimal sedation protocol required training in UCLA Health’s ambulatory nursing sedation protocols; this may differ at other institutions. Preliminary data using the minimal sedation protocol demonstrates a 50% success rate among individuals with IDD and/or needle-related anxiety [38]. UCLA Health’s minimal sedation guidelines, including pre-, during, and post-procedure care, are available for replication [38].

## Discussion

Individuals with IDD are at greater risk of poor physical health outcomes and needle-related anxiety, oftentimes attributable to a lack of accessible and inclusive healthcare. In order to improve health disparities for this population, more innovative strategies are required for them to receive quality primary care. The goal of this three-tier program was to provide easy-to-implement strategies in a patient-preferred healthcare setting. While most individuals will benefit from the behavior modifications in Tier 1 and the distraction and pain management techniques in Tier 2, the novel Tier 3 minimal sedation protocol enables equitable access to healthcare for anyone for whom routine needle procedures cannot be administered with lesser interventions.

Lessons learned

Among the multidisciplinary team who created the guidelines for the Needle Anxiety Program, some strategies were simpler to gain buy-in than others. Tier 1 and Tier 2 interventions are strongly supported in the literature [20-31], which allowed for universal approval to implement into the nurses’ training. Also, the topical numbing cream for the clinic was easy to obtain from the larger university-based hospital system. The integration of the Tier 3 minimal sedation protocol in an outpatient clinic required more extensive efforts to receive clearance from the university-based health system. Since the literature lacked a gold standard protocol for minimal sedation in the outpatient setting, UCLA Health’s ambulatory nursing team had safety concerns about implementing a novel protocol in the outpatient setting. However, the intranasal medication spray protocol was developed in collaboration with pharmacists and supported by anesthesiologists as an anxiety medication rather than sedation, which helped garner approval from the ambulatory nursing team. Another challenge was organizing the delivery of controlled substances to the outpatient clinic; facilities that wish to implement a similar protocol must have temperature-regulated and safely secured storage space before storing controlled substances. 

When implementing the minimal sedation protocol in outpatient settings, it is important to provide a clear definition of minimal sedation to nursing staff. Most people correlate sedation with sleep; yet, minimal sedation is an anxiety medication that helps patients relax during their visit and is often used in dentistry [34]. The protocol is straightforward, such that a novice or experienced RN can implement the program. In our program, two RNs administered the minimal sedation to patients: one RN has been a nurse for 20 years and the other nurse has been a nurse for less than five years. Both RNs participated in the minimal sedation protocol training and implemented the program successfully.

During the nurses’ training, there were additional concerns related to nurse safety. If the medical procedure is unable to be administered using evidence-based practices, patients should always be allowed to leave the appointment without completing the intended medical procedures. This strategy provides an opportunity for successful visits in the future. Additionally, the implementation of evidence-based behavioral strategies may reduce risks to healthcare providers by avoiding distress reactions that can arise when patients are restrained instead.

For other healthcare settings interested in adapting the Needle Anxiety Program, Tier 1 and Tier 2 evidence-based interventions are low-cost and effective techniques that are easy to implement and train nurses and other healthcare providers. Tier 1 and Tier 2 strategies can be utilized in various scenarios that include needle and non-needle-related procedures and improve the overall healthcare experience for patients with IDD. Depending on your institution there may be challenges to implementing a minimal sedation protocol (Tier 3) due to the capacity of the facility and the institution's bureaucracy. The facility must be capable of storing the intranasal medication spray in a temperature-regulated space and have enough nursing staff to assist with the protocol. Additionally, the minimal sedation protocol requires a physician to be on-site in case of an emergency. Lastly, a facility's institutional processes may require multiple department approvals, leading to delays or barriers in implementing the minimal sedation protocol. Overall, it's important to consider an institution's capacities when integrating the Needle Anxiety Program and consider modifications as needed.

The next steps for the Needle Anxiety Program include tracking the program’s outcomes and refining the program based on patients’ needs. A limitation of our methodology was not including individuals with IDD and their families in the formal development of this initiative. However, patient input from medical visits was informally included by physicians and nurses on the expert committee. Due to limited resources and in light of the COVID-19 pandemic, there was an immediate need for vaccinations which motivated the rapid development of the Needle Anxiety Program. As we continue to refine this program, we plan to include patients' and family members' input as experts in their own health experiences by involving a patient and family member advisory board. Another next step includes scaling the program to other clinics within the larger UCLA Health system; currently, the program is being administered at one community-based clinic. As we plan for greater implementation of the program, a cost-benefit analysis of the Needle Anxiety Program will be included. 

## Conclusions

The initial development and implementation of the Needle Anxiety Program has been well-received by healthcare providers and patients with IDD and/or needle-related anxiety. The three-tier program aims to reduce patient discomfort and increase the administration of routine needle-related medical procedures through: Tier 1 - training nurses in evidence-based behavioral interventions, Tier 2 - using distraction devices and pain management strategies, and Tier 3 - using a novel minimal sedation protocol. The overall goals of the Needle Anxiety Program are to reduce health inequities that arise from poor primary care outcomes for patients with IDD. Future work will focus on implementing a more rigorous evaluation of the feasibility and effectiveness of the program. We hope this paper can be an initial resource for other outpatient healthcare facilities implementing similar approaches to needle-related care of patients with IDD.
